# Chemotherapy Plus Radiotherapy *Versus* Chemotherapy Alone for Patients With Peripheral T-Cell Lymphoma, Not Otherwise Specified

**DOI:** 10.3389/fonc.2021.607145

**Published:** 2021-02-18

**Authors:** Zegeng Chen, He Huang, Xiaoqian Li, Xiaojie Fang, Zhao Wang, Huangming Hong, Zhihui Zhang, Qingqing Cai, Zhiming Li, Meiting Chen, Yuyi Yao, Fei Pan, Limin Chen, Tongyu Lin

**Affiliations:** ^1^ Department of Oncology, Sun Yat-sen University Cancer Center, State Key Laboratory of Oncology in Southern China, and Collaborative Innovation Center of Cancer Medicine, Guangzhou, China; ^2^ Department of Oncology, Sichuan Cancer Hospital, Chengdu, China

**Keywords:** radiation therapy, peripheral T-cell lymphoma, not otherwise specified, SEER, overall survival, disease-specific survival

## Abstract

**Purpose:**

Peripheral T-cell lymphoma, not otherwise specified (PTCL-NOS) is a clinically and biologically heterogeneous disease with poor prognosis. As the role of radiation therapy (RT) is still unclear, we carried out this study to evaluate the potential efficacy of RT in PTCL-NOS.

**Methods:**

Patients diagnosed with PTCL-NOS between 2000 and 2016 were identified from the Surveillance, Epidemiology, and End Results (SEER) database. Propensity score matching was used to balance the characteristics between patients who received radiotherapy and those who did not receive radiotherapy. In addition, we validated the findings in an external validation cohort retrospectively recruited from two high-capacity cancer center in China between 2006 and 2016. Kaplan-Meier curves and Cox regression models were used for survival analysis.

**Results:**

Of the 2,768 patients with chemotherapy records in the SEER cohort, 27.6% of 844 patients with early-stage disease and 6.8% of 1,924 patients with advanced-stage disease received RT. The application of RT was significantly associated with an improvement in overall survival (5-year OS rate 58.5 *versus* 35.1%, P <0.001) and disease-specific survival (5-year DSS rate 66.3 *versus* 44.0%, P <0.001) in the early-stage subgroup, while no apparent survival benefit of adding RT was identified in patients with advanced-stage disease (5-year OS rate 28.7 *versus* 24.4%, P = 0.089; 5-year DSS rate 32.9 *versus* 31.3%, P = 0.223). After adjustment, a matched cohort of 1,044 patients (348 in the RT combined with CT group and 696 in the CT alone group) was created. And RT was still significantly associated with a survival benefit in the early-stage subset, but not in the advanced-stage disease group. In the validation cohort with more comprehensive data, RT also significantly improved the survival of early-stage PTCL-NOS patients.

**Conclusion:**

Adding RT was associated with significant improvement in survival in early-stage PTCL-NOS, but the survival benefit of RT was not obvious in advanced-stage disease. The incorporation of RT for treatment in early-stage PTCL-NOS should be highly considered. Further prospective studies with more comprehensive data are needed to evaluate the effectiveness and toxicity of RT in PTCL-NOS.

## Introduction

Peripheral T-cell lymphoma, not otherwise specified (PTCL-NOS) is a histologically aggressive subtype of PTCL with widespread metastasis and poor clinical outcomes (1). In North America and Europe, PTCL-NOS is the most common subtype of PTCL (34.4% and 34.3% of PTCL, respectively), while the proportion is slightly lower in Asia (22.4% of PTCL) (2). In the United States, PTCL-NOS has an incidence of only 2,500 cases per year. Compared with B-cell lymphomas, PTCLs have not acquired the same degree of improvement in survival over the past 20 to 30 years (3). The intrinsic heterogeneity and its rarity have obstructed progress in this disease.

Without an optimal first-line treatment option, anthracycline-based regimens are mostly used in the induction treatment of PTCL-NOS patients, but they are rarely curative. It was reported that the complete response (CR) rate was between 17 and 70% in PTCL-NOS patients who received anthracycline-based regimens as an induction therapy (4, 5). Anthracycline-free combinations have also been tested as first-line therapy in previously untreated PTCL patients. In a phase 2 study published by Mary Gleeson *et al*. (6), the GEM-P (gemcitabine, cisplatin, and methylprednisolone) regimen was not superior to CHOP for the clinical outcome. In recent years, several studies have evaluated the efficacy of regimens that incorporate new agents effectively used in relapsed or refractory patients, but few of them showed significant improvement in survival to date (7–10).

Radiation therapy (RT) has been indicated to be an effective therapeutic strategy for many kinds of lymphoma, especially in early-stage diseases (11–14). For example, in another important subtype of T-cell lymphoma, extranodal natural killer/T-cell lymphoma (ENKTL), the addition of RT significantly improved clinical outcomes in early-stage disease, and RT alone resulted in a higher CR rate than chemotherapy alone in patients with localized ENKTL (2, 13, 14). Radiotherapy also resulted in higher response rates and provided additional survival benefits to patients with advanced-stage ENKTL (15). However, the value of RT in PTCL-NOS is still unclear, only a few retrospective studies with very small samples have been performed to explore it. In a retrospective review of 35 patients with stage I/II PTCL-NOS, RT combined with chemotherapy led to better outcomes than chemotherapy alone (16). It is vital to explore whether the application of RT can truly improve the poor prognosis of PTCL-NOS in a more convincing study. Therefore, we carried out this much larger sample study containing information from the US population-based Surveillance, Epidemiology, and End Results (SEER) cancer registries to evaluate the potential role of RT in improving the survival of patients with PTCL-NOS.

## Methods

### Patient Selection

This study was based on data from the SEER database. The database “SEER 18 Regs Custom Data with additional treatment field, Nov 2018 Sub (1975–2016 varying)” was searched for “2.2.1 Peripheral T-cell lymphoma, NOS”; patients aged 18 years or older diagnosed with PTCL-NOS between 2000 and 2016 were considered for study inclusion. Patients without complete survival data or with only autopsy or death certificate records were excluded from this study. Patients who did not definitively receive chemotherapy and those with an unknown stage were also excluded. The variable radiation therapy was classified as “beam radiation,” “combination of beam with implants or isotopes,” “radiation, NOS method or source not specified,” “radioactive implants (includes brachytherapy),” “radioisotopes,” “refused,” or “no/unknown” according to the SEER program. We defined “refused” and “no/unknown” as “no radiation therapy,” and the others as “radiation therapy.”

In addition to the radiotherapy status, the following covariates were analyzed in the SEER cohort: sex, age, race, Ann Arbor stage, extranodal involvement, and B symptoms. Since an increasing number of new agents have been used in the treatment of PTCL in recent years (7–10), a derived variable, whether primary diagnosis occurred before or after 2009, was also included because the treatment preferences may be different during the large time period.

The external validation cohort was consisted of PTCL-NOS patients who were diagnosed between January 2006 and December 2016 at Sun Yat-Sen University Cancer Center (SYSUCC) and Sichuan Cancer Hospital. Both the two hospitals are top cancer center in China with high capacity. Patients who met the inclusion criteria in this study were included.

All patients of the validation cohort received frontline chemotherapy, and some of them received involved-field radiotherapy (IFRT) in frontline or posterior line treatment. IFRT was delivered *via* 3-dimensional conformal, intensity-modulated, and electron-beam modalities and using 6MV-X ray generated by the linear accelerator. The target volume included all involved areas of disease at presentation (all involved lymph node sites and extranodal disease sites) with an adequate margin of at least 1 cm, according to the anatomic location. If safety was guaranteed, a 5 cm craniocaudal margin was also recommended.

### Statistical Analysis

Overall survival (OS) was determined by using the SEER vital status recode and was defined as the time from the initial diagnosis to the last follow-up date or the date of death from any cause. Another endpoint, disease-specific survival (DSS), was determined by using the SEER cause-specific death classification and COD (cause of death) to site recode and was defined as the time from diagnosis to death that was coded as being related to PTCL-NOS. Patients who were marked as alive or with any other cause of death were censored at the last follow-up date or the date of death. We described the characteristics grouped by treatment and evaluated univariate significant differences using chi-squared analysis. Propensity score matching (PSM), a widely accepted method to control for selection bias in observational studies (17), was used to create comparable cohorts of patients receiving chemotherapy alone and chemotherapy combined with RT on the basis of pretreatment characteristics. By using logistic regression models, the propensity score was determined with RT status as the dependent variable. The model was fit with covariates associated with the treatment or outcome, including sex, age, race, Ann Arbor stage, extranodal involvement, B symptoms, and diagnosis year. Propensity score 1:2 matching was used to pair each patient in the chemotherapy plus RT group with two patients in the group that received chemotherapy alone, whose propensity score was within the caliper width of 0.2. Standardized differences of means (SDM) were used to evaluate covariate balance, with SDM of <0.1 considered to be acceptable balance.

Kaplan–Meier curves were used to compare the survival of patients who received RT and those who did not receive RT, and a Cox proportional hazards model was used for multivariate survival analysis. After exploring the value of RT in the SEER data cohort, we validated the findings in the external validation cohort of patients who were recruited from China.

P<0.05 in a two-sided significance test was regarded as statistically significant. All statistical analyses were performed using R version 3.6.2 software (Institute for Statistics and Mathematics, Vienna, Austria).

## Results

### Patient Characteristics

**Figure 1:**
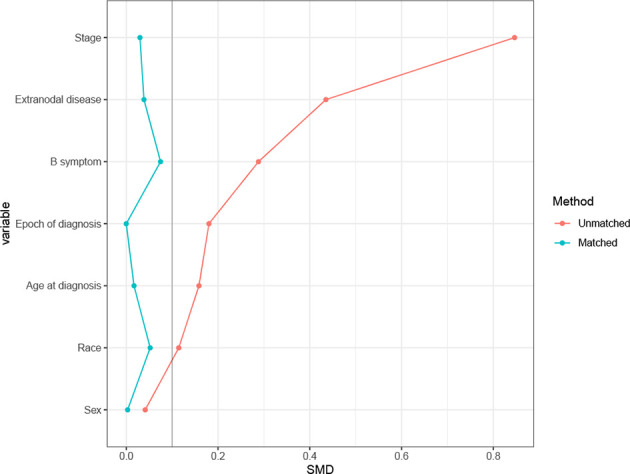
Absolute standardized differences of means (SDM) between treatment arms before and after the propensity score adjustment in Surveillance, Epidemiology, and End Results (SEER) cohort.

In the SEER cohort, a total of 2,768 cases were included. The median age of the patients was 62 (range 18–95) years, with 1,581 patients (57.1%) over 60 years old. Among 1,920 patients with B symptoms records, 849 patients (44.2%) had B symptoms at presentation, and the majority of patients had advanced-stage disease (Ann Arbor stages III/IV, n= 1,924, 69.5%). A minority of patients (n=363, 13.1%) received RT, with beam radiation as the most commonly used method (n=353, 97.2%). Before propensity score matching, patients with younger ages, early stage disease, B symptom absence, extranodal involvement, and diagnosis before 2009 were associated with a higher chance of receiving RT (**Table 1**). After matching, the variables affecting treatment selection or survival were adequately balanced, with SDM of all covariates less than 0.1 (**Figure 1**). Patients who did not have a proper match were excluded. Finally, a matched cohort of 1,044 patients (348 in the RT combined with CT group and 696 in the CT alone group) was used for subsequent analysis.

**Table 1:** Patient characteristics stratified by treatment in Surveillance, Epidemiology, and End Results (SEER) cohort.

Characteristic	Total	Treatment strata		P
	(n=2,768)	CT+RT (n=363)	CT alone (n=2,405)	
Sex				
Male	1,741 (62.9)	222 (61.2)	1,519 (63.2)	0.484
Female	1,027 (37.1)	141 (38.8)	886 (36.8)	
Age				
<60 years	1,187 (42.9)	185 (51.0)	1,002 (41.7)	0.001
>60 years	1,581 (57.1)	178 (49.0)	1,403 (58.3)	
Race/ethnicity				
White	2,044 (73.8)	271 (74.7)	1,773 (73.7)	0.126
Black	477 (17.3)	52 (14.3)	425 (17.7)	
Other	247 (8.9)	40 (11.0)	207 (8.6)	
Year of diagnosis				
2000–2008	1,409 (50.9)	213 (58.7)	1,196 (49.7)	0.002
2009–2016	1,359 (49.1)	150 (41.3)	1,209 (50.3)	
B symptoms				
Absence	1,071 (38.7)	166 (45.7)	905 (37.6)	<0.001
Presence	849 (30.7)	72 (19.8)	777 (32.3)	
Unrecorded	848 (30.6)	125 (34.4)	723 (30.1)	
Ann Arbor stage				
I/II	844 (30.5)	233 (64.2)	611 (25.4)	<0.001
III/IV	1,924 (69.5)	130 (35.8)	1,794 (74.6)	
Extranodal disease				
No	2,119 (76.6)	216 (59.5)	1,903 (79.1)	<0.001
Yes	649 (23.4)	147 (40.5)	502 (20.9)	

CT, chemotherapy; RT, radiation therapy.

#### Overall Survival Analysis

**Figure 2:**
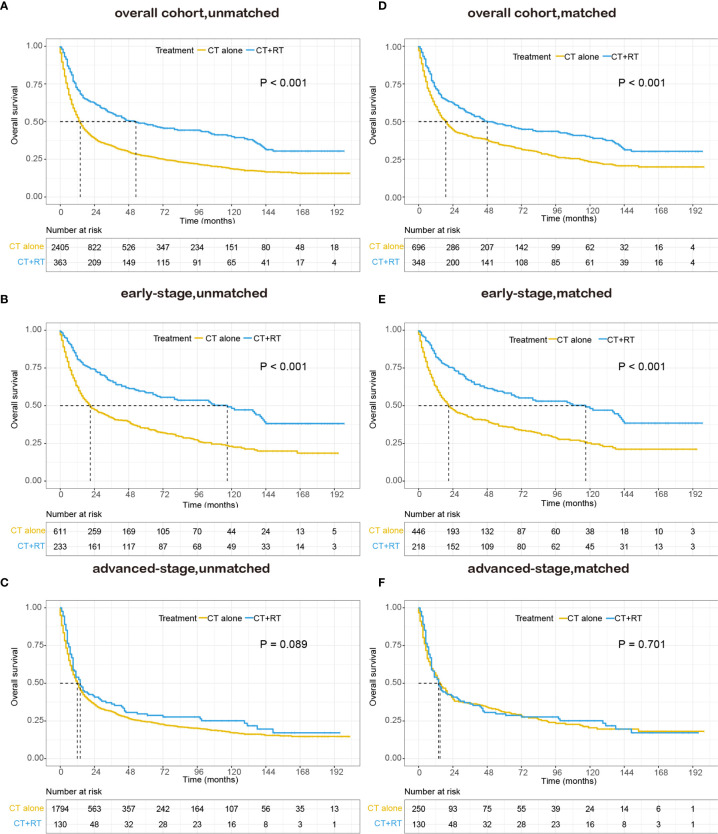
Kaplan–Meier survival curves of overall survival in patients with peripheral T-cell lymphoma, not otherwise specified (PTCL-NOS) stratified by RT administration status in the Surveillance, Epidemiology, and End Results (SEER) cohort: **(A)** unmatched overall cohort; **(B)** unmatched early-stage subgroup; **(C)** unmatched advanced-stage subgroup; **(D)** matched overall cohort; **(E)** matched early-stage subgroup; **(F)** matched advanced-stage subgroup; CT, chemotherapy; RT, radiation therapy.

In the unmatched cohort, with a median follow-up of 91.0 months, the 5-year OS rate in this study was 30.0% (95% CI: 28.2–31.8). Kaplan-Meier analysis demonstrated a significant improvement in OS (median 53 *versus* 14 months, 5-year survival rate 48.3 *versus* 27.2%, P < 0.001) in the chemotherapy plus RT group compared with the chemotherapy alone group (**Figure 2A**). We stratified patients into two analysis subgroups: early-stage (n=844) and advanced-stage (n=1,924). In the early-stage subgroup, the application of RT was still associated with a significant improvement in OS (median 117 *versus* 21 months, 5-year OS rate 58.5 *versus* 35.1%, P < 0.001) (**Figure 2B**), whereas in the advanced-stage subgroup, the benefit of the addition of RT was not significant (median 14 *versus* 12 months, 5-year OS rate 28.7 *versus* 24.4%, P = 0.089, **Figure 2C**). In the matched cohort, adding RT was still significantly associated with superior OS in the full cohort (median 48 *versus* 19 months, 5-year OS rate 47.7 *versus* 34.2%, P <0.001, **Figure 2D**) and the early-stage subset (median 117 *versus* 21 months, 5-year OS rate 58.4 *versus* 36.1%, P <0.001, **Figure 2E**). However, no apparent difference was observed in OS between patients who received RT and those who did not receive RT in the advanced-stage subset (median 14 *versus* 15 months, 5-year OS rate 28.7 *versus* 30.7%, P =0.701, **Figure 2F**).

#### Disease-Specific Survival Analysis

**Figure 3:**
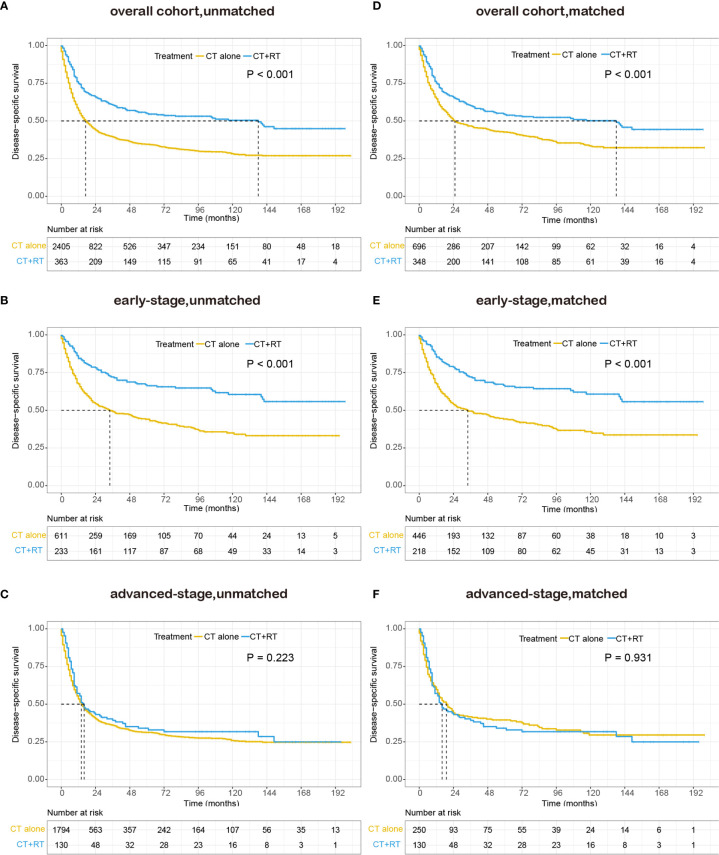
Kaplan–Meier survival curves of disease-specific survival in patients with peripheral T-cell lymphoma, not otherwise specified (PTCL-NOS) stratified by RT administration status in the Surveillance, Epidemiology, and End Results (SEER) cohort: **(A)** unmatched overall cohort; **(B)** unmatched early-stage subgroup; **(C)** unmatched advanced-stage subgroup; **(D)** matched overall cohort; **(E)** matched early-stage subgroup; **(F)** matched advanced-stage subgroup; CT, chemotherapy; RT, radiation therapy.

We also performed survival analysis of disease-specific survival, and the results were consistent with those of OS. In the unmatched cohort, Kaplan-Meier analysis demonstrated a significant improvement in DSS (median DSS 138 *versus* 17 months, 5-year DSS rate 55.0 *versus* 34.5%, P < 0.001) in the group that received RT compared with the group that did not receive RT (**Figure 3A**). In the early-stage subgroup, patients derived a significant survival benefit from the application of RT (median DSS not reached *versus* 34 months, 5-year DSS rate 66.3 *versus* 44.0%, P < 0.001, **Figure 3B**), whereas the benefit of the application of RT was not significant in the advanced-stage subgroup (median DSS 16 *versus* 14 months, 5-year DSS rate 32.9 *versus* 31.3%, P = 0.223, **Figure 3C**). In the matched cohort, the use of RT still significantly improved DSS in the full cohort (median 138 *versus* 25 months, 5-year DSS rate 54.2 *versus* 42.6%, P <0.001, **Figure 3D**) and the early-stage subset (median DSS not reached *versus* 34 months, 5-year DSS rate 65.9 *versus* 44.2%, P <0.001, **Figure 3E**). However, no obvious difference was observed in DSS between patients with RT and those without RT in the advanced-stage subset (median 16 *versus* 19 months, 5-year DSS rate 32.9 *versus* 39.3%, P =0.931, **Figure 3F**).

#### Multivariate Survival Analysis

In the multivariable Cox regression analysis of OS in the unmatched cohort, after adjusting for sex, age, race, diagnosis year, B symptoms, Ann Arbor stage, and extranodal involvement, RT was associated with significant improvement in OS (HR 0.663, 95% CI: 0.570–0.773, P <0.001). Early stage, younger age, more recent year of diagnosis, B symptom absence, and no extranodal involvement were also associated with superior OS in multivariate analysis (**Table 2**). In the early-stage subgroup, RT was still associated with a significant improvement in OS (HR 0.527, 95% CI: 0.427–0.651, P <0.001). However, in patients with advanced-stage disease, the benefit of RT did not reach statistical significance (HR 0.853, 95% CI: 0.690–1.054, P = 0.140, **Table 3**). The findings of the multivariable analysis of OS in the matched cohort were similar to those in the unadjusted cohort (**Table 4**). The application of RT significantly improved OS in both the full cohort (HR 0.684, 95% CI: 0.579–0.807, P <0.001) and the early-stage subset (HR 0.521, 95% CI: 0.416–0.652, P <0.001). However, no difference was observed in OS (HR 0.953, 95% CI: 0.738–1.232, P = 0.715) between patients who received RT and those who did not receive RT in the advanced-stage group.

**Table 2:** Cox regression model for multivariable analysis in Surveillance, Epidemiology, and End Results (SEER) cohort: overall survival (OS) and disease-specific survival (DSS).

Parameter	OS			DSS		
	HR	95% CI	P	HR	95% CI	P
Sex						
Male				Reference		
Female	1.005	0.917–1.102	0.913	0.998	0.901–1.105	0.972
Age						
<60 years				Reference		
>60 years	1.722	1.568–1.891	<0.001	1.605	1.447–1.780	<0.001
Race/ethnicity						
White				Reference		
Black	1.072	0.950–1.210	0.258	1.121	0.983–1.279	0.088
Other	1.119	0.953–1.312	0.169	1.211	1.020–1.437	0.029
Year of diagnosis						
2000–2008				Reference		
2009–2016	0.884	0.800–0.977	0.015	0.865	0.775–0.964	0.009
B symptoms						
Absence				Reference		
Presence	1.224	1.095–1.368	<0.001	1.204	1.066–1.361	0.003
Unrecorded	1.107	0.988–1.240	0.080	1.087	0.959–1.233	0.192
Ann Arbor stage						
I/II				Reference		
III/IV	1.490	1.334–1.664	<0.001	1.594	1.408–1.805	<0.001
Extranodal disease						
No				Reference		
Yes	1.192	1.065–1.335	0.002	1.142	1.007–1.296	0.039
Treatment						
CT alone				Reference		
CT+RT	0.663	0.570–0.773	<0.001	0.660	0.556–0.784	<0.001

OS, overall survival; DSS, disease-specific survival; CI, confidence interval; HR, hazard ratio; CT, chemotherapy; RT, radiation therapy.

**Table 3:** Multivariate subgroup survival analysis of overall survival (OS) and disease-specific survival (DSS) in Surveillance, Epidemiology, and End Results (SEER) cohort.

Parameter	OS				DSS			
	Stage I/II		Stage III/IV		Stage I/II		Stage III/IV	
	HR (95% CI)	P	HR (95% CI)	P	HR (95% CI)	P	HR (95% CI)	P
Sex								
Male	Reference							
Female	0.950 (0.793–1.138)	0.578	1.021 (0.917–1.137)	0.703	0.963 (0.783–1.183)	0.718	1.008 (0.897–1.134)	0.889
Age								
<60 years	Reference							
>60 years	2.116 (1.754–2.554)	<0.001	1.603 (1.439–1.787)	<0.001	1.897 (1.534–2.346)	<0.001	1.497 (1.330–1.684)	<0.001
Race/ethnicity								
White	Reference							
Black	1.065 (0.823–1.379)	0.632	1.073 (0.936–1.231)	0.311	1.119 (0.837–1.497)	0.449	1.114 (0.962–1.291)	0.150
Other	0.969 (0.704–1.335)	0.849	1.188 (0.987–1.429)	0.069	1.111 (0.785–1.573)	0.552	1.250 (1.026–1.522)	0.027
Year of diagnosis								
2000–2008	Reference							
2009–2016	0.814 (0.664–0.997)	0.047	0.913 (0.814–1.024)	0.122	0.757 (0.616–0.931)	0.008	0.905 (0.800–1.024)	0.115
B symptoms								
Absence	Reference							
Presence	1.398 (1.101–1.775)	0.006	1.161 (1.023–1.318)	0.020	1.283 (0.974–1.691)	0.076	1.163 (1.015–1.334)	0.030
Unrecorded	1.146 (0.927–1.416)	0.208	1.107 (0.973–1.259)	0.123	1.129 (0.887–1.437)	0.324	1.099 (0.955–1.264)	0.189
Extranodal disease								
No	Reference							
Yes	1.157 (0.971–1.379)	0.103	1.164 (1.006–1.346)	0.042	1.170 (0.958–1.428)	0.124	1.086 (0.921–1.280)	0.328
Treatment								
CT alone	Reference							
CT+RT	0.527 (0.427–0.651)	<0.001	0.853 (0.690–1.054)	0.140	0.479 (0.373–0.614)	<0.001	0.904 (0.721–1.134)	0.383

OS, overall survival; DSS, disease-specific survival; CI, confidence interval; HR, hazard ratio; CT, chemotherapy; RT, radiation therapy.

**Table 4:** Multivariate survival analysis of overall (OS) and disease-specific survival (DSS) in propensity score matched cohorts-radiation therapy.

	OS			DSS		
	HR	95% CI	P	HR	95% CI	P
Overall cohort (n=1,044)	0.684	0.579–0.807	<0.001	0.674	0.559–0.813	<0.001
Early-stage group (n=664)	0.521	0.416–0.652	<0.001	0.472	0.363–0.614	<0.001
Advanced-stage group (n=380)	0.953	0.738–1.232	0.715	1.004	0.760–1.326	0.979

OS, overall survival; DSS, disease-specific survival; HR, hazard ratio; CI, confidence interval.

In the multivariable Cox regression analysis of DSS in the unmatched cohort, RT was significantly associated with superior DSS (HR 0.660, 95% CI: 0.556–0.784, P <0.001, **Table 2**). In the early-stage subgroup, RT was still associated with a significant improvement in DSS (HR 0.479, 95% CI: 0.373–0.614, P <0.001). However, the benefit of RT did not reach statistical significance in patients with advanced-stage disease (HR 0.904, 95% CI: 0.721–1.134, P = 0.383, **Table 3**). The results of the multivariable analysis of DSS in the matched cohort were consistent with those in the unmatched cohort (**Table 4**). Adding RT was still a good prognostic factor in all patients (HR 0.674, 95% CI: 0.559–0.813, P <0.001) and the early-stage subset (HR 0.472, 95% CI: 0.363–0.614, P <0.001). However, RT was no longer a prognostic factor in the multivariable analysis of DSS in the advanced-stage group (HR 1.004, 95% CI: 0.760–1.326, P = 0.979).

#### Validation Cohort

In the validation cohort, 143 patients were identified at two high-capacity hospitals in China between 2006 and 2016. The clinical characteristics of the 143 patients are summarized in **Table 5**. Similar to the results of SEER cohort, most of patients had advanced-stage disease (n=98, 68.5%), and extranodal involvement was very common (n=96, 67.1%). The median age was 53 years old (range: 18–91), with 38 patients (26.6%) over 60 years old. In regard to the distribution of the IPI scores, a majority of patients (n=99, 69.2%) had IPI scores between 0 and 2.

**Table 5:** Patient characteristics stratified by disease staging in validation cohort.

Characteristic	Total	Ann Arbor stage	
	(n=143)	I/II (n=45)	III/IV (n=98)
Sex			
Male	100 (69.9)	31 (68.9)	69 (70.4)
Female	43 (30.1)	14 (31.1)	29 (29.6)
Age			
<60 years	105 (73.4)	30 (66.7)	75 (76.5)
>60 years	38 (26.6)	15 (33.3)	23 (23.5)
Serum lactate dehydrogenase			
Normal	86 (60.1)	31 (68.9)	55 (56.1)
Elevated	57 (39.9)	14 (31.1)	43 (43.9)
ECOG PS			
0/1	103 (72.0)	34 (75.6)	69 (70.4)
≥2	40 (28.0)	11 (24.4)	29 (29.6)
B symptoms			
Absence	92 (64.3)	32 (71.1)	60 (61.2)
Presence	51 (35.7)	13 (28.9)	38 (38.8)
Extranodal disease			
No	47 (32.9)	20 (44.4)	27 (27.6)
Yes	96 (67.1)	25 (55.6)	71 (72.4)
Bone-marrow involvement			
No	126 (88.1)	45 (100)	81 (82.7)
Yes	17 (11.9)	0	17 (17.3)
IPI score			
0/1	57 (39.9)	33 (73.3)	24 (24.5)
2	42 (29.4)	11 (24.4)	31 (31.6)
3	29 (20.3)	1 (2.2)	28 (28.6)
4/5	15 (10.5)	0	15 (15.3)
Radiation therapy			
No	113 (79.0)	27 (60.0)	86 (87.8)
Frontline therapy	22 (15.4)	16 (35.6)	6 (6.1)
Posterior line therapy	8 (5.6)	2 (4.4)	6 (6.1)

ECOG PS, Eastern Cooperative Oncology Group performance status; IPI, International Prognostic Index.

All the patients received chemotherapy in frontline treatment, among various chemotherapy regimens, CHOP was the most commonly used (n=57, 58.8%), following by CHOEP as the second most common first-line chemotherapy regimen (n=42, 29.4%). The other chemotherapy regimens were used based on the condition of patients and the physicians’ experience.

In the early-stage subgroup, 16 patients (35.6%) received frontline radiation therapy, and another two patients received palliative RT when they experienced a progression of disease. While in the advanced-stage subgroup, only six patients (6.1%) received RT in frontline treatment and six patients (6.1%) received posterior line RT. Radiation therapy was administered to patients with a median dose of 45 Gy (range, 24–55 Gy), at a dose per fraction of 2 Gy.

**Figure 4:**
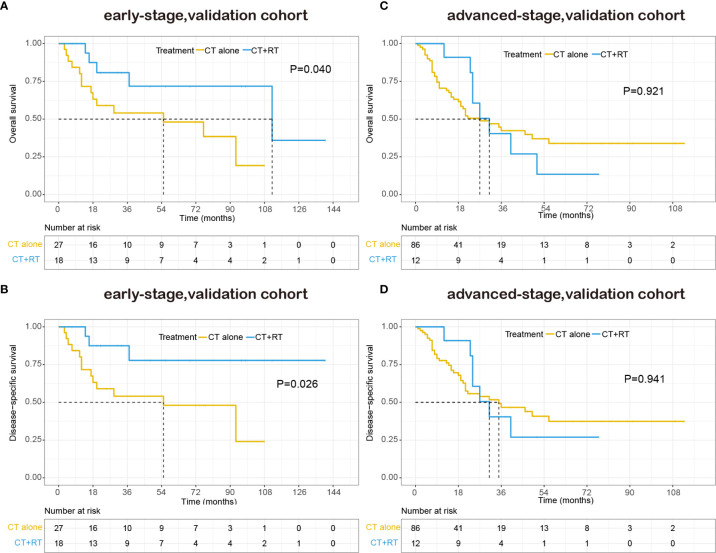
Kaplan–Meier survival curves of overall survival and disease-specific survival in patients with peripheral T-cell lymphoma, not otherwise specified (PTCL-NOS) stratified by RT administration status in the validation cohort: **(A)** early-stage, overall survival; **(B)** early-stage, disease-specific survival; **(C)** advanced-stage, overall survival; **(D)** advanced-stage, disease-specific survival.

With a median follow-up of 48.0 months, 70 patients (49.0%) had died, 85.7% of them died of lymphoma or treatment-related complications. The 5-year OS and DSS for all patients were 39.9% and 44.1%, respectively. The Kaplan–Meier survival curves of overall survival and disease-specific survival in the validation cohort are shown in **Figure 4**. In the early-stage subgroup, among 41 patients with response evaluation records, 19 patients (46.3%) achieved CR after frontline treatment. For the 16 patients received CT combined with RT as frontline treatment, 11(68.8%) of them achieved CR, and 4 (25%) of them achieved partial remission (PR), only one patient (6.3%) showed stable disease (SD) after treatment. However, a much lower CR rate (32.0%) was achieved after CT alone in frontline therapy as compared with those (68.8%) with CT plus RT (P=0.029). And eight (32.0%) of 25 patients received CT alone showed SD or progressive disease (PD) after treatment. Among 87 advanced-stage patients with response evaluation records, 35 patients (40.2%) achieved CR after first-line treatment. Six patients received CT plus RT in first-line treatment, two (33.3%) of them achieved CR, and two (33.3%) of them achieved PR, the other two patients (33.3%) experienced a progression of disease after treatment. There was no significant difference in CR rate between CT plus RT group and CT alone group (33.3 *vs*. 40.7%, P=1.000).

The patient characteristics were analyzed for prognostic value on OS and DSS. On multivariate analysis, extranodal involvement > 2 sites was the only independent prognostic factor that significantly associated with OS (HR 1.718, P=0.045). The adding of RT also showed a trend toward better OS (HR 0.595, P=0.095) and DSS (HR 0.524, P=0.063) on multivariate analysis. As is shown in **Table 6**, in the early-stage subgroup, frontline RT was a significant prognostic factor for OS (HR 0.277, P=0.045) and DSS (HR 0.198, P=0.034), while posterior line RT was not a prognostic parameter for OS or DSS on multivariate analysis. What’s more, in the advanced-stage subgroup, neither frontline RT nor posterior line RT was associated with OS and DSS.

**Table 6:** Multivariate subgroup survival analysis of overall survival (OS) and disease-specific survival (DSS) in validation cohort.

Parameter	OS				DSS			
	Stage I/II		Stage III/IV		Stage I/II		Stage III/IV	
	HR (95% CI)	P	HR (95% CI)	P	HR (95% CI)	P	HR (95% CI) P	
Sex								
Male	Reference							
Female	0.735 (0.254–2.125)	0.570	2.016 (1.120–3.631)	0.019	0.889 (0.291–2.718)	0.837	1.768 (0.972–3.218)	0.062
Age								
<60 years	Reference							
>60 years	0.737 (0.255–2.127)	0.572	1.427 (0.763–2.668)	0.266	0.355 (0.080–1.579)	0.174	1.457 (0.693–3.064)	0.321
ECOG PS								
0/1	Reference							
≥2	1.115 (0.318–3.905)	0.865	0.671 (0.340–1.325)	0.251	0.902 (0.182–4.474)	0.899	0.590 (0.279–1.250)	0.169
Serum LDH								
Normal	Reference							
Elevated	1.265 (0.451–3.553)	0.655	1.208 (0.671–2.176)	0.529	1.422 (0.463–4.361)	0.539	1.149 (0.613–2.156)	0.665
Bone-marrow involvement								
No	Reference							
Yes	–	–	1.238 (0.587–2.613)	0.575	–	–	1.176 (0.515–2.682)	0.700
B symptoms								
Absence	Reference							
Presence	1.594 (0.576–4.414)	0.369	1.692 (0.952–3.007)	0.073	2.018 (0.658–6.192)	0.220	1.578 (0.860–2.895)	0.141
Extranodal involvement								
0–1	Reference							
>1	1.459 (0.265–8.033)	0.664	1.848 (1.037–3.293)	0.037	1.366 (0.156–11.990)	0.778	1.481 (0.788–2.784)	0.223
Radiation therapy								
No	Reference							
Frontline	0.277 (0.079–0.974)	0.045	1.818 (0.685–4.825)	0.230	0.198 (0.044–0.886)	0.034	1.786 (0.677–4.714)	0.242
Posterior line	0.734 (0.095–5.661)	0.766	0.697 (0.213–2.285)	0.552	0.761 (0.098–5.891)	0.793	0.492 (0.116–2.083)	0.335

OS, overall survival; DSS, disease-specific survival; CI, confidence interval; HR, hazard ratio; ECOG PS, Eastern Cooperative Oncology Group performance status; LDH, lactate dehydrogenase.

## Discussion

PTCL-NOS is a histologically aggressive disease with poor clinical outcomes that frequently has extranodal disease involvement and advanced stages (1, 18). In a study carried out by the International T-Cell Lymphoma Project, the 5-year OS rate for patients diagnosed with PTCL-NOS between January 1990 and December 2002 was 32% (19). In this study with a large cohort of patients, the 5-year OS rate was 30.0% (95% CI: 28.2–31.8), which confirms that there has been no significant survival improvement in recent years, though new first-line combination regimens have been tried. And we found that combination therapy with chemotherapy and RT was associated with superior OS compared with chemotherapy alone in PTCL-NOS patients (5-year OS 48.3 *versus* 27.2%, P < 0.001).

The data of this study was acquired from the SEER database and hospitals in China, so the age distribution was more representative of the real world (median age of 62 years old in the SEER cohort and 53 years old in the validation cohort), with patients with higher levels of comorbidities and non-cancer cause of death risks than those in randomized trials. Therefore, in addition to OS, another important study endpoint, DSS, was used in the survival analysis to balance the potential bias caused by non-cancer causes of death. We found that the application of RT was associated with superior DSS in PTCL-NOS patients.

In the subgroup analysis stratified by disease stage, the survival benefit of the application of RT was significant in stage I-II patients. In the validation cohort with more comprehensive data, it was the frontline RT but not the posterior line RT that improve the survival of early-stage PTCL-NOS patients. However, only 27% of patients with early-stage disease received RT in the SEER cohort. If chemotherapy was performed as the primary therapy for localized PTCL-NOS patients, radiation therapy, especially frontline RT, should be highly considered for these patients. On the other hand, no apparent survival benefit of adding RT was identified in patients with advanced-stage disease. The probable explanation for this finding is that RT results in excellent locoregional control, while it has limited effectiveness in advanced-stage patients with widespread lesions. It is important to continue to explore whether RT is a prognostic factor in select situations involving advanced-stage disease in further studies that contain larger sample with more detailed information, including clinical characteristics and treatment options.

The details of chemotherapy, including chemotherapy regimens and number of cycles, are not available in the SEER database. However, as anthracycline-based chemotherapy regimens are considered to be standard treatment in frontline treatment in recent decades, the CHOP regimen has been applied in a proportion of patients ranging from 60 to 85% (2, 20, 21). It was consistent with the results of validation cohort that CHOP and CHOEP were applied to 88.2% PTCL-NOS patients as frontline chemotherapy regimens. Therefore, we speculated that the majority of patients in the SEER cohort were likely to receive relatively similar chemotherapy regimens in the contemporary era (from 2000 to 2016). To minimize treatment selection bias inherent in retrospective studies, propensity score matching analysis was carried out in the SEER cohort. And the findings of the matched cohort were totally consistent with those of the unadjusted cohort, which suggested RT was an independent prognostic factor in PTCL-NOS.

Extranodal involvement >1 site and Ann Arbor stage III/IV were important prognostic factors in the International Prognostic Index (IPI) (22). However, these two factors were not included in the prognostic index for peripheral T-cell lymphoma unspecified (PIT) because they lost independent prognostic value in multivariate analysis for PIT (23). In our study using a larger cohort of patients with PTCL-NOS in the SEER database, Ann Arbor stage III/IV and extranodal involvement were significantly associated with inferior OS and DSS in multivariate analysis. In the validation cohort, extranodal involvement >1 site was also an independent prognostic factor on OS. Since some important prognostic factors, including lactate dehydrogenase (LDH) levels, performance status and bone marrow status, were not included in the multivariate analysis in the SEER cohort, we believe it is of great importance to re-evaluate the predictive value of these commonly used prognostic factors in future studies with more comprehensive data.

In contrast to previous reports that recruited patients with a small set of samples (2, 16, 21), our study contains an adequate sample size to explore differences in clinical characteristics and survival for patients with PTCL-NOS. To our knowledge, this is the only study to date that specifically explored the potential role of RT in patients with both early-stage and advanced-stage disease. Moreover, as these data were collected from 18 registries covering approximately 34.6% of the US population, biases due to referral and lower access to care were notably reduced compared with those in trials performed at single or few multiple institutions.

There are several limitations in our study. First, some important prognostic features, including LDH levels, performance status and bone marrow status (23), are not available within the SEER database. Although we controlled for selection bias and potential confounders of the survival benefit using propensity score adjustment. These unavailable features may significantly influence the likelihood of RT administration and the subsequent clinical outcome. Another important limitation is the ambiguous record of not receiving RT (no RT/unknown-no evidence of radiation was found in the medical records examined) within the SEER database. Noone AM et al. compared treatment determined by SEER with therapeutic strategies collected by Medicare claims and indicated that the overall sensitivity and positive predictive value of the SEER data for the radiation variable were approximately 80% and high (>85%), respectively (24). It is inevitable that some patients in the CT alone group might have received RT, which may have influenced their survival. Additionally, the information about RT treatment planning is very finite in the SEER database. We were unable to ascertain the RT technologies, target volumes, or radiotherapy dose. However, we believe that the heterogeneity of RT treatment planning was probably somewhat mitigated because of the contemporary era this study used. In order to remedy the limitations above, a validation cohort recruited from China with more comprehensive data were included in this study. We did find that RT significantly improved OS and DSS of patients with early-stage PTCL-NOS in the validation cohort.

In conclusion, we found that patients with early-stage PTCL-NOS who received chemotherapy treatment had a significant survival benefit from RT, while no obvious survival difference was observed between patients who received RT and those who did not receive RT in the advanced-stage group. Therefore, the potential role of incorporating RT for treatment in early-stage disease should be highly considered. Future prospective trials with more comprehensive data are needed to evaluate the effectiveness and toxicity of RT in PTCL-NOS.

## Data Availability Statement

Publicly available datasets were analyzed in this study. This data can be found here: https://seer.cancer.gov/data/.

## Ethics Statement

Ethical review and approval was not required for the study on human participants in accordance with the local legislation and institutional requirements. Written informed consent for participation was not required for this study in accordance with the national legislation and the institutional requirements.

## Author Contributions

ZC and TL designed the study. HH, XL, ZW, XF, HMH, ZZ, QC, and ZL contributed to data collecting and analysis. ZC wrote the initial draft of the manuscript. HH and XL wrote sections of the manuscript. TL, MC, YY, FP, XL, and LC reviewed and edited the manuscript. All authors contributed to the article and approved the submitted version.

## Funding

This study was supported by Guangdong Science and Technology Department (grant 2017B020227002).

## Conflict of Interest

The authors declare that the research was conducted in the absence of any commercial or financial relationships that could be construed as a potential conflict of interest.
